# The Expression of a Subset of Aging and Antiaging Markers Following the Chondrogenic and Osteogenic Differentiation of Mesenchymal Stem Cells of Placental Origin

**DOI:** 10.3390/cells13121022

**Published:** 2024-06-12

**Authors:** Mahmoud Zhra, Ahmad M. Magableh, Lara M. Samhan, Lein M. Fatani, Rani J. Qasem, Ahmad Aljada

**Affiliations:** 1 Department of Biochemistry and Molecular Medicine, College of Medicine, Alfaisal University, Riyadh 11533, Saudi Arabia; 2 College of Medicine, Alfaisal University, Riyadh 11533, Saudi Arabia; 3 Department of Pharmacology and Pharmacy Practice, College of Pharmacy, Middle East University, Amman 11831, Jordan

**Keywords:** *LMNA/C*, SIRT7, SM22α, mesenchymal stem cells, phenotypic drift, osteocytes, chondrocytes, adipocytes

## Abstract

Mesenchymal stem cells (MSCs) of placental origin hold great promise in tissue engineering and regenerative medicine for diseases affecting cartilage and bone. However, their utility has been limited by their tendency to undergo premature senescence and phenotypic drift into adipocytes. This study aimed to explore the potential involvement of a specific subset of aging and antiaging genes by measuring their expression prior to and following in vitro-induced differentiation of placental MSCs into chondrocytes and osteoblasts as opposed to adipocytes. The targeted genes of interest included the various *LMNA/C* transcript variants (lamin A, lamin C, and lamin A∆10), sirtuin 7 (SIRT7), and SM22α, along with the classic aging markers plasminogen activator inhibitor 1 (PAI-1), p53, and p16^INK4a^. MSCs were isolated from the decidua basalis of human term placentas, expanded, and then analyzed for phenotypic properties by flow cytometry and evaluated for colony-forming efficiency. The cells were then induced to differentiate in vitro into chondrocytes, osteocytes, and adipocytes following established protocols. The mRNA expression of the targeted genes was measured by RT-qPCR in the undifferentiated cells and those fully differentiated into the three cellular lineages. Compared to undifferentiated cells, the differentiated chondrocytes demonstrated decreased expression of SIRT7, along with decreased PAI-1, lamin A, and SM22α expression, but the expression of p16^INK4a^ and p53 increased, suggesting their tendency to undergo premature senescence. Interestingly, the cells maintained the expression of lamin C, which indicates that it is the primary lamin variant influencing the mechanoelastic properties of the differentiated cells. Notably, the expression of all targeted genes did not differ from the undifferentiated cells following osteogenic differentiation. On the other hand, the differentiation of the cells into adipocytes was associated with decreased expression of lamin A and PAI-1. The distinct patterns of expression of aging and antiaging genes following in vitro-induced differentiation of MSCs into chondrocytes, osteocytes, and adipocytes potentially reflect specific roles for these genes during and following differentiation in the fully functional cells. Understanding these roles and the network of signaling molecules involved can open opportunities to improve the handling and utility of MSCs as cellular precursors for the treatment of cartilage and bone diseases.

## 1. Introduction

Mesenchymal stem cells (MSCs) hold great promise in the field of regenerative medicine and tissue engineering. The multipotent nature of these cells allows them to differentiate into various cellular lineages, which include chondrocytes and osteocytes. The inherent ability of MSCs to differentiate into cartilage made them an attractive therapeutic approach to stimulate articular cartilage repair and regeneration [1,2]. In addition, their ability to differentiate into osteocytes qualified them for the treatment of osteoporosis. However, the utility of these cells has been challenged by their tendency to undergo premature senescence and phenotypic drift into adipocytes, which may happen in vitro during expansion, or in vivo following transplantation [2,3,4]. Therefore, there is a need for a better understanding of the molecular mechanisms involved in the maintenance of these cells before, during, and after differentiation because it can provide novel insights that can help overcome these shortcomings and improve the clinical utility of these cells for the treatment of cartilage and bone diseases.

Several proteins are known for their role in cellular senescence and have been investigated in the differentiation of MSCs into various cell types. One prominent example is lamin A/C proteins. These are fibrous nuclear proteins that provide structural support for the nuclear architecture. They also propagate extracellular mechanical and biological signals and interact with transcription factors [5]. Generally, the increased accumulation of lamin A/C proteins is regarded as a sign of cellular senescence, in addition to the fact that the expression of these proteins has been relatively absent in stem cells [6,7]. Recent experimental evidence indicates that the increased expression of p53, a known inducer of cellular senescence, mediates its effect through the stabilization of lamin A/C proteins and the subsequent stimulation of p16^INK4a^, which is also a recognized inducer of cellular aging [7]. In addition to its potential role in senescence, the expression of lamin A/C proteins can also influence the differentiation of stem cells including those mesenchymal in origin [8,9]. For example, Zhang et al. demonstrated that *LMNA/C* haploinsufficiency, which causes a 50% reduction in gene expression, impaired the osteogenic differentiation of mouse embryonic stem cells [10]. In yet another investigation, the suppression of lamin A/C expression encouraged adipogenic differentiation in a rat mesenchymal progenitor cell line [11]. The role of lamins in influencing the fate of stem cells has been described in the recent review by Zhang et al. [12]. Although lamin A/C proteins are the two primary transcript variants of the *LMNA/C* gene and the focus of most investigations, two other less abundant variants of the same gene are found in mammalian somatic cells. These are lamin AΔ10 and lamin C2; the latter is only produced in the testis. Generally, the various variants of the *LMNA/C* gene have been measured as a single protein in previous investigations, and the role of each variant in the differentiation of MSCs is currently unknown.

Sirtuins (SIRTs) and plasminogen activator inhibitor-1 (PAI-1) are also known regulators of cellular senescence that can influence the differentiation of MSCs. Sirtuins (SIRTs) are nicotine adenine dinucleotide (NAD+)-dependent histone deacetylases that regulate metabolism and DNA repair and help protect against many of the aging-associated pathologies such as oxidative stress and inflammation. Studies have confirmed that various types of SIRTs play a crucial role in enhancing longevity and promoting differentiation of MSCs. For example, SIRT1 promoted the chondrogenic differentiation of MSCs through the inhibition of NF-κB and the activation of Sox9 [13]. The disruption of SIRT1 function like in the case of oxidative stress encouraged adipogenic over osteocytic lineage commitment [14]. Likewise, SIRT3, which functions as a mitochondrial deacetylase that protects against oxidative stress without affecting oxidative metabolism, was shown to improve longevity and stimulate the differentiation of MSCs into adipocytes and osteocytes [15]. Among the SIRT proteins, SIRT7 is the least studied, but studies have associated a decrease in its expression with several age-related processes [16,17]. The expression of SIRT7 is reported to limit the differentiation of bone marrow-derived MSCs into osteocytes through the inhibition of the Wnt/β–catenin signaling pathway [18]. PAI-1 is a member of the superfamily of serine-protease inhibitors and is the primary mechanism that inhibits plasminogen activation. The expression of PAI-1 is significantly increased with aging, and it has been associated with a range of age-related subclinical and clinical pathologies [19,20,21]. It is also a stress-regulated gene, and its induction increases the risk for thrombosis in older populations [22]. The expression of PAI-1 is vital for the differentiation of MSCs into osteocytes [20]. However, its expression has also been associated with decreased survival of MSCs in vivo [23]. Finally, the smooth muscle protein-alpha of 22kDa (SM22α), also known as TAGLN or Transgelin, has been described to arrest growth and promote cellular senescence through the p16^INK4a^ pathway [24,25]. It has been implicated in vascular diseases, as its accumulation accelerated the senescence of vascular smooth muscle cells by stabilizing p53 [24]. 

In this investigation, we examined the expression profiles for the various *LMNA/C* transcript variants (lamin A, lamin C, and lamin A∆10), SIRT7, PAI-1, SM22α, p53, and p16^INK4a^ in the undifferentiated MSCs and following the in vitro-induced differentiation of the cells into chondrocytes, osteocytes, and adipocytes. Our hypothesis is that the differentiation process is associated with unique changes in the expression of these genes in the three cellular lineages. The exploration of the mechanisms involved in the differential expression of these genes and the molecular network involved should improve our understanding of the performance of MSCs in vitro during expansion and following differentiation, which can open opportunities to exploit through pharmacologic interventions to improve the performance of these cells in vitro and in vivo with greater precision.

## 2. Materials and Methods

### 2.1. Isolation and Culture MSCs

The placentas used in this investigation were obtained from uncomplicated pregnancies with normal vaginal delivery of male births between 38 and 40 weeks of gestation. Informed consent was obtained from all women according to the guidelines approved by King Abdullah International Medical Center (KAIMC) and King Abdulaziz Medical City (KAMC). The placenta was processed within two hours after delivery, and MSCs were isolated from decidua basalis as described previously [26]. In brief, 10 g of decidua tissue was dissected, washed with phosphate-buffered saline (PBS), minced, and then centrifuged at 300× *g* for 5 min. The tissue was then digested with 0.3% collagenase type I (Life Technology, Carlsbad, CA, USA) and diluted in PBS, along with 100 µg/mL streptomycin, 100 U/mL penicillin, and 271 U/mL DNase I (Life Technology, Carlsbad, CA, USA) for a duration of 1 h in a water bath set at a temperature of 37 °C. The resulting mixture was filtered through a 100 μm nylon filter (Becton Dickinson, Franklin Lakes, NJ, USA), followed by centrifugation and incubation for 45 min with a red blood cell lysing buffer (#sc-3621, FCM Lysing solution, Santa Cruze, Dallas, TX, USA). Subsequently, the cells were centrifuged, washed, and cultured in T25 flasks in a complete MSC culture medium (DMEM-F12) supplemented with 10% fetal bovine serum, 100 µg/mL L-glutamate, 100 µg/mL streptomycin, and 100 U/mL penicillin. The cells were incubated at a temperature of 37 °C in a humidified atmosphere consisting of 5% CO_2_ and 95% air. 

### 2.2. Flow Cytometry

MSCs were evaluated for phenotypic characteristics by flow cytometry between passages 3 and 5 as described previously [26]. Briefly, cells were detached using a 0.05% trypsin solution and subsequently stained with monoclonal antibodies for 30 min at room temperature. After two washes with cold PBS, the cells were centrifuged at 150× *g* for 5 min at 4 °C. Subsequently, the cells were fixed with 4% paraformaldehyde in sterile PBS, pH 7.4, for 10 min at room temperature and permeabilized with 0.1% saponin for 5 min. The immunoreactivity of the cell surface antibody markers or intracellular proteins was then analyzed using a BD FACS CANTO II flow cytometer (Becton Dickinson, Franklin Lakes, NJ, USA).

### 2.3. Colony-Forming Unit (CFU) Assay

The colony-forming unit of the cells was evaluated as previously described [26]. The cells were seeded at a rate of 100 cells per well into six-well plates with a complete culture medium. The cells were fixed using 4% paraformaldehyde for 30 min at room temperature, followed by staining with 0.1% crystal violet for 15 min and subsequent rinsing with distilled water. Colonies, defined as clusters comprising 50 or more cells, were observed and quantified under a microscope.

### 2.4. Osteogenic, Chondrogenic, and Adipogenic Induction

The isolated and cultured MSCs were induced to differentiate into osteogenic, chondrogenic, and adipogenic lineages following established procedures [26]. Basically, the cells were seeded into six-well plates at a density of 10^4^ cells per well and cultured in a complete MSC medium supplemented with an osteogenic supplement for osteogenic differentiation (StemXVivo^®^ human osteogenic supplement, old part # 390416, current catalog # CCM008, R&D systems, Minneapolis, MN, USA) or with a chondrogenic supplement for chondrogenic differentiation (StemXVivo^®^ human chondrogenic supplement, old part # 390417, current catalog number CCM006, R&D systems, Minneapolis, MN, USA) or with an adipogenic supplement for adipogenic differentiation (StemXVivo^®^ human adipogenic supplement, old part # 390415, current catalog number CCM011, R&D systems, Minneapolis, MN, USA). Each one of these media contains high-quality differentiation factors that allow for the reproducible differentiation of the MSCs into the respective cellular lineages. The culture medium was refreshed every 3 days. After 21 days of culture, the differentiated osteocytes, chondrocytes, and adipocytes were washed twice with PBS and then collected for RNA isolation. Cellular differentiation was confirmed by phase-contrast microscopy for morphological features and staining as described previously [26], with HCS LipidTOX Green neutral lipid stain for adipocytes, Alizarin Red S for osteocytes, and Alcian Blue for chondrocytes. A comparison was made between the differentiated cells and the original undifferentiated MSCs cultured in a medium lacking the forgoing supplements.

### 2.5. RT-qPCR

To determine the mRNA expression level for SIRT7, PAI-1, *LMNA/C* transcript variants (lamin A, lamin C, and lamin AΔ10), p53, SM22α, and p16^INK4a^, the total RNA was extracted using an Ambion Aqueous Kit (Ambion, Inc., Austin, TX, USA), and the quality and quantity of the extracted RNA were assessed using the Agilent Bioanalyzer automated electrophoresis system (Santa Clara, CA, USA). For reverse transcription, 1 μg of total RNA was converted to cDNA using an Advantage RT-for-PCR Kit (Clontech; Mountain View, CA, USA). Real-time RT-qPCR was performed on the Applied Biosystems 7900HT platform (Foster city, CA, USA). Taqman assays were conducted with 2 μL of cDNA, 10 μL Taqman Master Mix (Qiagen; Valencia, CA, USA), and 0.5 μL of 20 μM gene-specific primers and 5′ 6-FAM/3′ BHQ-1^®^ dual-labeled probes for lamin A, lamin C, lamin AΔ10, and SIRT7 (Table 1). Sybergreen RT-qPCR was performed to quantitate other aging/antiaging proteins using 2 µL cDNA, 10 µL 2X Sybergreen Master Mix, and 0.5 µL of 20 μM gene-specific primers (Table 1) obtained from Bio Basic Canada Inc. (Markham, Ontario, Canada). The PCR protocol consisted of an activation cycle of 50 °C for 2 min, followed by 95 °C for 15 s. Subsequent amplification ensued with 40 cycles of denaturation at 95 °C for 15 s and annealing/extension at 60 °C for 2 min. Although normalization to ribosomal protein L13a (RPL13a), Ubiquitin C, GAPDH, and cyclophilin A showed similar trends, all values were ultimately normalized to cyclophilin A for consistency. The 2^−ΔΔCT^ technique was utilized to perform relative quantification in qRT-PCR. The basal mRNA expression of the markers in the MSCs was adjusted to a standard of 0. An elevation in expression is indicated by positive values, while a reduction is represented by negative values [27].

### 2.6. Statistical Analysis

All of the results presented herein are mean ± standard error of the mean (SEM). All cultures and all mRNA analyses were performed in triplicate. Statistical analysis was performed using the SigmaStat software version 3.0 (Jandel Scientific, San Rafael, CA, USA). All data were ensured to pass the normal distribution and equal variance tests prior to statistical testing. A paired *t*-test was applied to compare the basal values to fold change in mRNA expression after the induced differentiation of the cells. A *p* < 0.05 was considered statistically significant.

## 3. Results

**Figure 1:**
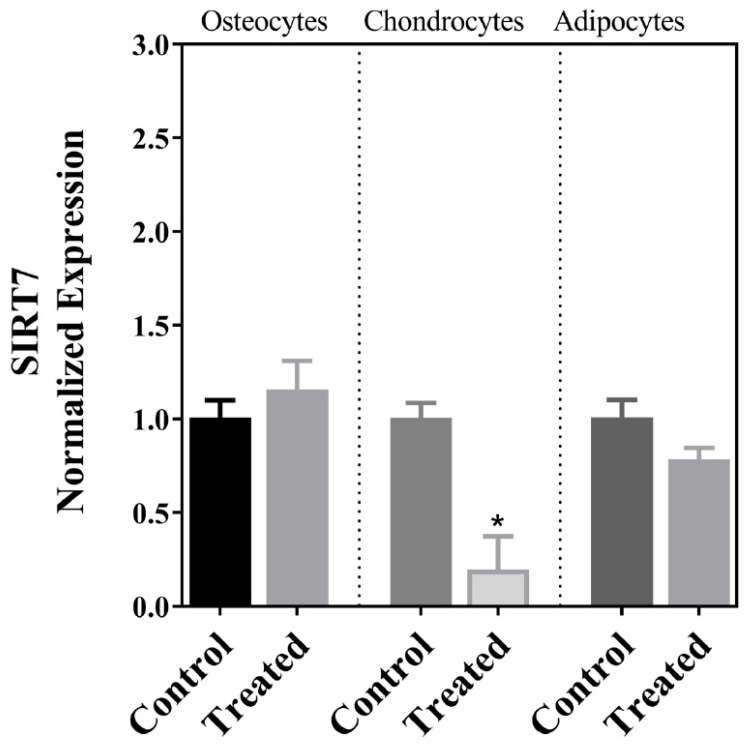
SIRT7 expression as quantitated by RT-qPCR in undifferentiated placental MSCs (control) and cells following in vitro-induced osteogenic, chondrogenic, and adipogenic differentiation. Statistically significant findings are annotated with an asterisk (*).

**Figure 2:**
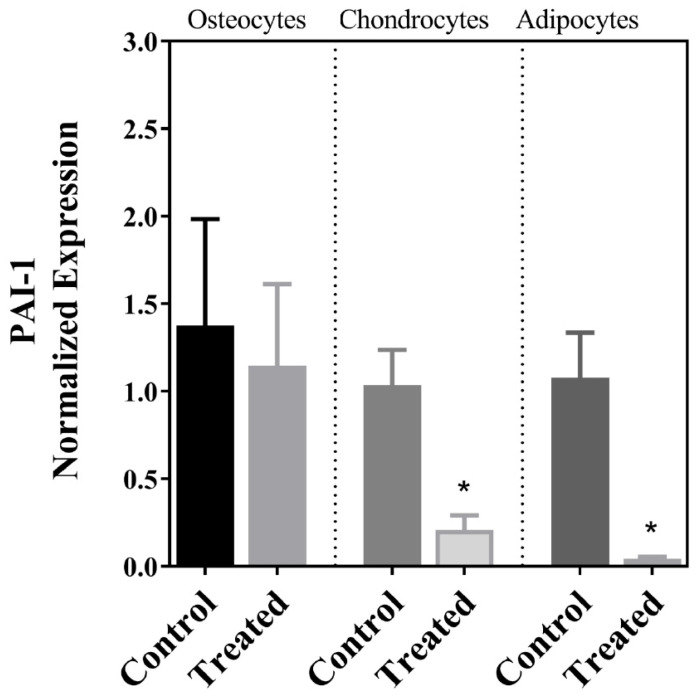
The expression of PAI-1 in undifferentiated placental MSCs (control) and cells following in vitro-induced osteogenic, chondrogenic, and adipogenic differentiation. Statistically significant findings are annotated with an asterisk (*).

**Figure 3:**
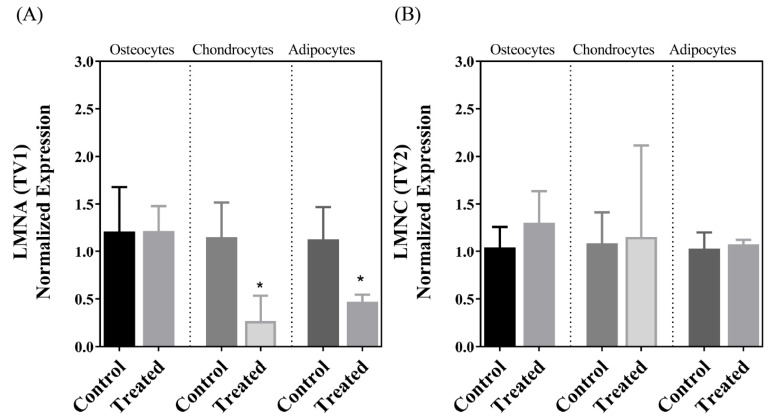
(**A**) LMNA and (**B**) LMNC expression in undifferentiated placental MSCs (control) and cells following in vitro-induced osteogenic, chondrogenic, and adipogenic differentiation. Statistically significant findings are annotated with an asterisk (*).

**Figure 4:**
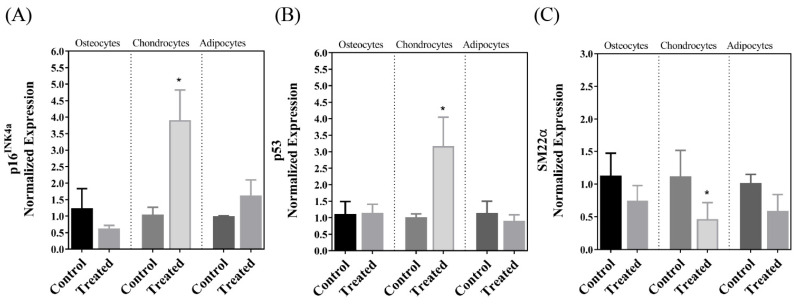
Expression of (**A**) p16^INK4a^, (**B**) p53, and (**C**) SM22α in undifferentiated placental MSCs (control) and cells following in vitro-induced osteogenic, chondrogenic, and adipogenic differentiation. Statistically significant findings are annotated with an asterisk (*).

The MSCs isolated from the decidua basalis of the human term placenta passed the quality criteria for purity, clonogenic capability, markers, and differentiation into chondrocytes, osteocytes, and adipocytes within 21 days following induction, and the results of these purity and functional evaluations and staining of the cells following differentiation have been previously published [26]. Our analyses of gene expression in this investigation indicated that there was a significant decrease in the expression of SIRT7 after the differentiation of MSCs into chondrogenic cells but not following osteogenic or adipogenic differentiation (Figure 1). This was accompanied by a parallel decrease in the expression of PAI-1, which also occurred after adipogenic differentiation (Figure 2). The expression pattern of the lamin A transcript in the three cellular lineages mirrored the expression pattern of PAI-1, so it decreased after chondrogenic and adipogenic differentiation but not after osteogenic differentiation (Figure 3A). On the other hand, no significant change in the expression of the lamin C transcript was detected following the differentiation of MSCs into the three cell types (Figure 3B). The expression of the LMNAΔ10 transcript variant was undetectable in the undifferentiated and differentiated MSCs. The decrease in SIRT7 expression during the chondrogenic differentiation of MSCs was accompanied by a noticeable increase in the expression of p53 and p16^INK4a^. Interestingly, the mRNA transcript levels of p53 and p16^INK4a^ did not change after osteogenic and adipogenic differentiation (Figure 4A,B). Finally, SM22α was detected in the undifferentiated MSCs, which is congruent with previous studies [28]. The expression of SM22α decreased following differentiation into the three cellular lineages but reached statistical significance only following chondrogenic differentiation (Figure 4C). A summary of the upregulation or downregulation of gene expression during the differentiation of MSCs into the three cellular lineages is provided in Table 2.

## 4. Discussion

MSC-based therapy represents an attractive option in regenerative medicine and tissue engineering for the treatment of cartilage and bone diseases. This stems from the inherent ability of these cells to transform into chondrocytes and osteocytes, respectively. In addition, the cells are known for the relative ease of isolation and in vitro expansion along with their immune-privileged and tumorigenic-free natures. The differentiation of MSCs into chondrocytes and osteocytes is a complex process that is influenced by a variety of factors. The differentiation of MSCs and their commitment to specific cellular lineages is influenced by an intricate balance in gene expression. Genes that promote or oppose aging can affect the differentiation of MSCs and shift them into adipocytes instead and/or induce premature senescence [1,2,3,4]. In the present investigation, we explored the expression profile of several aging and antiaging genes before and after in vitro-induced differentiation of MSCs into chondrocytes, osteocytes, and adipocytes, in an attempt to identify variations in expression that can be explored to improve the handling of these cells in vitro during expansion and their utility in vivo for the treatment of bone and cartilage diseases.

Perhaps the most intriguing finding in this investigation was the decrease in expression of SIRT7 following the differentiation of the cells into chondrocytes but not into osteocytes or adipocytes. In one previous investigation, SIRT-7 deficiency was required for the differentiation of bone marrow-derived MSCs into osteocytes, which occurred along with the activation of the Wnt/β–catenin signaling pathway and the simultaneous expression of the OSX transcription factor [18]. The maintained expression of SIRT7 following osteogenic differentiation in the present study as opposed to the previous investigation [18] suggests molecular and cellular differences between MSCs of bone marrow and those that are of placental origin. Previous investigations have reported differences between bone marrow and placental MSCs that include transcriptomic, proteomic, and architectural properties, along with factors that encourage these differences, which include the heterogenic nature of the isolated MSCs and differences in culturing conditions [29,30,31]. The results of this investigation corroborate the notion that differences between bone marrow and placental MSCs are not restricted to isolated cells but also include the expression profile of specific genes following differentiation, which can have a direct impact on the functional properties and the longevity of these cells in a graft. Because the SIRT family of proteins is known for promoting longevity and protection against oxidative stress, and that includes SIRT7 [16], it is conceivable that the maintained expression of SIRT7 following osteogenic differentiation in the present study is an advantage to MSCs of placental origin, especially considering the numerous functions SIRT7 plays in stabilizing the genome and regulating mitochondrial function [16]. On the other hand, it is conceivable that the decrease in expression of SIRT7 only following chondrogenic differentiation in the present investigation is a sign of the commitment of these cells to chondrocytes, more so than a sign of senescence. Previous investigations have associated the maintained expression of SIRT7 with cellular quiescence [16], so the decrease in its expression found in this investigation indicates that the MSCs enter a stage of cellular growth and differentiation. Notably, the decrease in SIRT7 expression was associated with a concomitant decrease in the expression of PAI-1, a marker of cellular stress and accelerated senescence [22]. It also accompanied a decrease in the expression of SM22α, which was also shown to induce senescence in various cell types [24,25]. In addition, the loss of SM22α has also been associated with the chondrogenic differentiation of vascular smooth muscle cells [32,33]. Collectively, these findings further corroborate our notion that the fall in SIRT7 expression is probably a marker for the chondrogenic differentiation of the cells. However, it is also our impression that MSCs differentiating into chondrocytes undergo aging at a rate faster than cells induced to differentiate into osteocytes and adipocytes. This is evident in the expression of p53 and p16^INK4a^ that increased only following chondrogenic differentiation. Previous investigations have associated the increased expression of p53 with the decreased proliferation and differentiation of MSCs [34], the increased apoptosis of chondrogenic cells [35], and the prognosis of osteoarthritis [36]. Similarly, p16^INK4a^ is a potent inhibitor of cell cycle progression, and an increase in its expression has been shown to correlate with pro-inflammatory molecules, collectively known as the senescence-associated secretory phenotype (SASP), and to be associated with chondrocyte dysfunction [37]. Alternatively, the increase in expression of these two genes can be a specific feature associated with cells that undergo chondrogenic differentiation. The specific mechanisms through which p53 and p16^INK4a^ modulate chondrogenic differentiation and their molecular interactions in this regard remain to be elucidated. Interestingly, in the present study, we did not observe a drop in the expression of p53 following osteogenic differentiation. The loss of p53 expression is reported to mark the osteogenic differentiation of MSCs that are of bone marrow origin, and it is believed to underlie the increase in the incidence of osteosarcoma [38,39]. Again, the disparity of our findings with previous studies can be explained by potential differences in the behavior and functional properties of MSCs of different origins [29,30,31]. The maintained expression of p53 levels in the differentiated osteogenic and adipogenic cells works against the possibility that these cells underwent senescence during in vitro culture in the present investigation.

Lamin A and C proteins are the two primary transcript variants of the *LMNA/C* gene. A minor variant known as lamin AΔ10 is also expressed in tissues. Studies indicate that the relative expression of each transcript variant—and the ratio among them—is determined in a cell/tissue-specific manner that can decide the structural integrity and mechnoelastic properties [40]. Investigators have reported different biological roles for the lamin transcript variants, despite the high similarity in primary amino acid sequences [41]. In addition, the ratio of the various variants can influence the cellular mechanoelastic properties and response to mechanical cues [40]. Although the lamin A protein is believed to exert important functions during development through its role as a sensor to the external and internal cellular environments, recent studies indicate that the lamin C protein has the strongest correlation with the mechanophenotypic properties of the cell [42]. The role of the lamins—collectively as a single protein—in MSC differentiation has been extensively investigated and reviewed by Zhang et al. Studies have shown that MSCs respond to mechanical stimulation and various forms of cellular stress by decreasing the levels of lamin A, which has been considered a sign of cellular senescence [43]. Generally, previous investigations indicate that the expression of lamin A/C proteins is required for osteogenic differentiation, while their suppression promotes the adipogenic differentiation of MSCs [11,44]. However, there has not been a description of the role of each one of these two lamins in the differentiation process. Research evidence suggests that the different mutations in the *LMNA/C* gene that are involved in laminopathies differentially affect the efficiency of MSCs to differentiate, with each mutation encouraging the expression of a specific set of genes that encourage the lineage-specific differentiation of MSCs [9]. Accordingly, in the present investigation, we determined the expression profile of the various *LMNA/C* transcript variants (lamin A, lamin C, and lamin A∆10) to determine whether the distinct patterns of expression for the variants reflect different roles and are associated with the lineage-specific differentiation of MSCs. It is noteworthy that the two primary variants of the *LMNA/C* gene, lamin A and lamin C, are considered interchangeable and are traditionally quantified and reported as a single protein. This reporting of the total expression of the gene products can obscure the unique contribution that individual transcript variants may have to cellular differentiation and mask essential insights into cellular processes. Interestingly, lamin A was the only variant that decreased in its expression following the differentiation of MSCs and occurred only following differentiation into chondrocytes and adipocytes. The expression levels of the protein were maintained after osteogenic differentiation. This finding is in line with the roles known for the lamin A protein during development and in aging. In contrast, the expression of lamin C remained constant following the differentiation of MSCs into the three cellular lineages, suggesting that this lamin variant has important functions after differentiation. The expression of the lamin A∆10 transcript variant was not detected in MSCs and after differentiation in the three cellular lineages, suggesting that it is irrelevant to the process. Therefore, it is concluded from these results that both lamins A and C contribute to the mechanoelastic features and structural integrity of the fully differentiated osteogenic cells, but only lamin C is responsible for these cellular properties in chondrogenic and adipogenic cells.

Cellular differentiation and aging are intimately associated aspects of cellular biology. Aging is known to intensify many pathological pathways such as oxidative and metabolic stresses, telomere attrition, and inflammation. In addition, aging is associated with weakened DNA repair and epigenetic changes. Collectively, these processes influence the renewal potential of stem cells and their ability to differentiate into specific cellular lineages, increasing the possibility of phenotypic drift during differentiation into the unintended phenotype [45,46,47]. On the other hand, experimental evidence indicates that cellular differentiation can expose several latent features of aging [48]. Perhaps one prominent example of the close relationship between these two aspects of cellular biology has been the finding that bone marrow MSCs of old age—as opposed to younger cells—exhibited reduced renewal potential and an increasing preference to differentiate into adipocytes instead of osteocytes, which is believed to be one of the factors underlying age-associated bone loss [45,46]. Several signaling cascades have been implicated in this observation. For example, the loss of the Notch signaling cascade, dysregulation in the Sirt1/FoxO/β-catenin pathway, and decreased levels of NAD^+^ can all decrease the expression of osteogenic markers, such as collagen and osteocalcin, and reduce the commitment of MSCs to develop into osteocytes [49,50]. These occur as other pathways that encourage adipogenesis are activated, such as the PPARγ2 signaling pathway [51]. In the present investigation, the transcript levels of none of the targeted genes changed following osteogenic differentiation of placental MSCs; however, a decrease in the expression of lamin A and PAI-1 occurred following adipogenic differentiation, suggesting that the decreased expression of these gene products may facilitate the path for adipogenesis. This conclusion corroborates findings in the literature; for example, the depletion of lamin A/C in MSCs encouraged adipogenic differentiation, while their enrichment increased osteogenic differentiation in one study [52], although it is important to note that this study, like many others, did not distinguish the contribution each of the individual lamins had to the findings. Likewise, a previous investigation has shown that PAI-1 is required for the expression of osteogenic markers in adipose and bone marrow-derived stem cells [20]. In addition, the decrease in the expression of PAI-1 augmented the adipogenic differentiation of pluripotent embryonic stem cells [53]. Understanding the molecular interactions in the background with established signaling pathways that promote osteogenic and adipogenic differentiation and the molecular outreach of our findings can provide important new insights on bone homeostasis and mechanisms of age-related osteoporosis and can help identify newer targets for treating bone diseases.

## 5. Conclusions

The regulation of MSC differentiation is a complex process involving various signaling pathways and transcription factors. Unraveling the pathways involved in the differentiation process holds profound significance on the clinical utility of these cells in regenerative medicine. The present investigation used in vitro models to explore the effects of several aging and antiaging genes on the differentiation of MSCs of placental origins into chondrocytes, osteocytes, and adipocytes. This investigation reports unique changes in the expression of SIRT7, lamin A vs. lamin C, PLA1, SM22α, p53, and p16^INK4a^ following the differentiation of MSCs into the respective cellular lineages. The differences in the expression of these genes reflect specific roles during and after differentiation in the fully functional specialized cells. Although the mRNA transcript levels have been conventionally used in the literature to reflect the expression of the genes analyzed in the present investigation, which is reflective of a reliable correlation between the transcript and protein levels, a definitive appreciation of their role in the differentiation process requires future investigations examining the functional properties of the protein products in relation to other well-established regulators and markers of MSC differentiation. Understanding the roles these gene products play in the differentiation process and their interactions with other molecular entities should help establish a comprehensive overview of the signaling cascades involved in the differentiation process and could unveil molecular pathways that can be pharmacologically manipulated to improve the differentiation of these cells in vitro and control over their fate in tissue engineering protocols and also their clinical utility in vivo in the treatment of cartilage and bone loss diseases.

## Figures and Tables

**Table 1:** Primer sequences used in RT-qPCR for the target genes.

Primer	Sense (5′→3′)	Antisense (5′→3′)	Probe (5′→3′)	Accession Number	Amplicon Length (bp)	Melting Temperature (°C)
SIRT7	ACTGCTTCAGAAAGGGAGA	CACAGTTCTGAGACACCACA	ACTGCTTCAGAAAGGGAGA	NM_016538.2	128	92.5
LMNA	TGACTGTGGTTGAGGACGAC	GACACTGGAGGCAGAAGAGC	CGCTGAGTACAACCT	NM_170707.3	221	98.5
LMNC	GTGGAAGGCACAGAACACCT	GCGGCGGCTACCACTCAC	AGATGACCTGCTCCATCACC	NM_005572.3	178	94.0
LMNAΔ10	AACTCCACTGGGGAAGGCTCC	GCTCCTGAGCCGCTGGCAGA	AGTACAACCTGCGCTCGCGC	NM_170708.3	131	98.0
p16^INK4a^	GGGGGCACCAGAGGCAGT	GGTTGTGGCGGGGGCAGTT		NM_000077.4	159	92
p53 (TP53)	CCGGCGCACAGAGGAAGAGA	TGGGGAGAGGAGCTGGTGTTGT		NM_000546.5	108	92.5
PAI-1	GTGTTTCAGCAGGTGGCGC	CCGGAACAGCCTGAAGAAGTG		NM_001386460.1	300	94.0
SM22α	TGGCGTGATTCTGAGCAA	CTGCCAAGCTGCCCAAGG		NM_001001522.2	239	92.5
Ubiquitin C	ACTACAACATCCAGAAAGAGTCCA	CCAGTCAGGGTCTTCACGAAG		NM_021009.6	85	88.0
RPL13	AACAAGTTGAAGTACCTGGCTTTC	TGGTTTTGTGGGGCAGCATA		NM_012423.4	130	95.3
Cyclophilin A	CCCACCGTGTTCTTCGACAT	TTTCTGCTGTCTTTGGGACCTT		NM_021130.5	94	92.0
GAPDH	ACCACAGTCCATGCCATCAC	TCCACCACCCTGTTGCTGTA		NM_002046.7	452	95.5

**Table 2:** Summary of the expression results for the target genes following the differentiation of the MSCs of placental origin into chondrocytes, osteocytes, and adipocytes. (↔): no change; (↓): inhibition, (↑): upregulation; (ND): none detected.

	Chondrogenic Lineage	Osteogenic Lineage	Adipogenic Lineage
SIRT7	↓	↔	↔
PAI-1	↓	↔	↓
LMNA	↓	↔	↓
LMNC	↔	↔	↔
LMNAΔ10	ND	ND	ND
p16^INK4a^	↑	↔	↔
p53 (TP53)	↑	↔	↔
SM22α	↓	↔	↔

## Data Availability

The data that support the findings reported in this investigation are available upon request from the corresponding author.
